# Artificial Intelligence in Dental Treatment Planning and Diagnostic Decision‐Making: A Systematic Review and Meta‐Analysis

**DOI:** 10.1002/cre2.70343

**Published:** 2026-03-31

**Authors:** Mohammad Alabdulkareem, Momen Atieh, Ammar AbuMostafa, Khaled Aldalaan, Nada Alturki

**Affiliations:** ^1^ College of Medicine and Dentistry, Department of Restorative Dentistry Riyadh Elm University Riyadh Saudi Arabia; ^2^ Mohammed Bin Rashid University of Medicine and Health Sciences, Hamdan Bin Mohammed College of Dental Medicine Dubai United Arab Emirates; ^3^ Sir John Walsh Research Institute, Faculty of Dentistry University of Otago Dunedin New Zealand; ^4^ School of Dentistry The University of Jordan Amman Jordan; ^5^ King Abdullah specialized Hospital, National Guard Qassim Saudi Arabia; ^6^ General Dentist, Private Dental Practice Riyadh Saudi Arabia

**Keywords:** artificial intelligence, clinical decision‐making, deep learning, dental diagnostics, dental imaging, diagnostic accuracy, machine learning, treatment planning

## Abstract

**Objectives:**

This systematic review and meta‐analysis aimed to synthesize the available evidence on the use of AI in dental diagnostic decision‐making and treatment planning, evaluating both diagnostic accuracy and its influence on clinical decision‐making across different dental specialties and imaging modalities.

**Methods:**

A comprehensive search of MEDLINE, Embase, Cochrane CENTRAL, Web of Science, and Scopus was conducted from database inception to December 2025. Eligible studies evaluated AI algorithms used for dental diagnostic tasks or treatment planning and reported quantitative performance metrics or measurable decision‐making outcomes. Random‐effects meta‐analyses were conducted to pool diagnostic performance measures.

**Results:**

Twenty‐seven studies involving 60,857 radiographic images were included. AI systems demonstrated a pooled sensitivity of 0.85 (95% CI: 0.76–0.91) and specificity of 0.94 (95% CI: 0.86–0.97). The pooled F1‐score was 0.90 (95% CI: 0.77–0.96), and pooled precision was 0.88 (95% CI: 0.71–0.96). For segmentation tasks, the pooled Dice Similarity Coefficient was 0.89 (95% CI: 0.13–1.00). Substantial heterogeneity was observed across studies (I² > 95%). YOLO‐based architectures achieved the highest performance for tooth detection and segmentation, with sensitivities approaching 99% and mean average precision exceeding 0.96. AI assistance also improved diagnostic efficiency and interobserver agreement while reducing diagnostic interpretation time.

**Conclusions:**

AI systems demonstrate strong diagnostic performance in dental imaging and decision support, particularly for tooth detection, segmentation, and pathology identification. However, substantial heterogeneity, retrospective study designs, and limited external validation highlight the need for rigorous prospective evaluation before widespread clinical implementation.

## 1 Introduction

Artificial intelligence represents a disruptive technology in healthcare, with dental medicine representing one of the fields where AI diagnostic and treatment planning tools are adopted particularly fast (Panahi 2025). The integration of machine learning algorithms, specifically deep learning architectures, including convolutional neural networks, has demonstrated significant promise in automating complex visual recognition tasks that have traditionally relied on extensive clinical expertise (Taye 2023). In dentistry, diagnosis highly relies on radiographic interpretation and visual assessment (Chauhan et al. 2023). AI systems are increasingly utilized for caries detection, the assessment of periodontal disease, the identification of periapical lesions, orthodontic land marking, and many other multiple specialty treatment planning decisions (Yigitaliev et al. 2025).

It is rightly said that the benefits of AI in dentistry are enormous (Panahi 2025). Imaging data can be analyzed using AI algorithms over a large volume of data without significant variation in performance, potentially limiting inter‐observer inconsistency that has long been a thorn in the flesh of dental diagnostic (Musleh et al. 2024). AI‐aided interpretation of dental pathology can help identify it at an early stage, potentially saving a patient, simplifying and decreasing the cost of treatment (Yigitaliev et al. 2025). Moreover, AI may be used to combat staffing shortages, as it can help increase the number of clinicians, including in underserved regions, where the availability of specialist knowledge is low (Balakrishnan et al. 2025). The introduction of dental imaging technologies to replace conventional two‐dimensional radiography with cone beam computed tomography (CBCT) and digital intraoral photography (Shah 2014) has significantly expanded the amount and volume of the information that needs to be interpreted, which has led to the emergence of an environment where AI support can be of special use.

Regardless of such optimism, the scientific foundation of AI application in the dental practice is still piecemeal and heterogeneous (de Magalhães and Santos 2025). Different studies assessing the performance of AI have diverse designs, validation methods, and outcome measures (Myllyaho et al. 2021). Internal validation is the basis of many studies, which hinders the extrapolation of the study to real‐life clinical practice (Blonde et al. 2018). The sample sizes tend to be small, and the range of patients and imaging regimens among studies makes it difficult to compare (Balki et al. 2019). Moreover, although many studies present impressive diagnostic accuracy scores of AI algorithms, a smaller number of them have tested whether these systems can actually enhance clinical decision‐making when they are incorporated into clinical practices (Castaneda et al. 2015). The important difference between algorithmic performance in controlled research and clinical usefulness in practice has not been well taken care of (Schuetz et al. 2011).

Several systematic reviews have focused on specific dental subspecialties, such as the detection of caries or orthodontic analysis, in which AI applications have been used (Thurzo et al. 2022). No comprehensive synthesis, however, has combined the evidence of AI applications across all dental diagnostic and treatment planning tasks with the evaluation of both diagnostic accuracy and impact on clinical decision‐making. This represents an especially important gap, because translation of diagnostic accuracy to changes in clinical outcomes is contingent on how AI systems affect practitioner behavior, quality of decisions, and, ultimately, patient care (Shamszare et al. 2025). These relationships can be comprehended only through synthesis across various study designs, including diagnostic accuracy studies, randomized controlled trials, and observational studies of clinician‐AI interaction.

Several important methodological questions remain unexamined. No trials have reported the comparative performance of different AI architectures systematically, the influence of validation methods on reported accuracy, and the modification of imaging modality and dental specialty on the effectiveness of AI. Publication bias––especially the failure to publish studies with null or unfavorable results––distorts the apparent promise of these technologies (Song et al. 2010). The quality of reference standards to train and validate AI systems also varies significantly; whereas some studies use single expert opinions, others use consensus panels or histopathological confirmation (Nagendran et al. 2020). Such methodological variations may cause large variations in the reported performance metrics, limiting the reliability of individual study findings.

This systematic review and meta‐analysis will perform a comprehensive, rigorous synthesis of the best available evidence on AI applications in dental diagnostic decision‐making and treatment planning. We will use robust diagnostic test accuracy meta‐analytic methods that account for the correlation between sensitivity and specificity to derive pooled estimates of the performance of AI in a wide range of clinical applications. In a systematic assessment of decision‐making outcomes, we are interested in determining whether AI systems meaningfully enhance diagnostic accuracy and clinical decision‐making in dental practice, with particular emphasis on radiographic interpretation across key dental specialties. Pre‐specified subgroup analyses will be performed by the following factors: imaging modality, dental specialty, AI model architecture, and validation approach, thereby guiding both future research and implementation. Finally, applying GRADE methodology for assessing evidence certainty will allow us to transparently communicate the strength and limitations of current evidence, enabling informed decisions about AI adoption in dental practice.

## 2 Methods

### 2.1 Protocol Registration and Reporting

This systematic review and meta‐analysis is registered in the International Prospective Register of Systematic Reviews, PROSPERO (Registration number: CRD420251240547), and was conducted in accordance with the Preferred Reporting Items for Systematic Reviews and Meta‐Analyses (PRISMA) 2020 guidelines (O'Dea et al. 2021), the Preferred Reporting Items for Systematic Reviews and Meta‐analyses of Diagnostic Test Accuracy Studies (PRISMA‐DTA) (McInnes et al. 2018), and the Cochrane Collaboration's recommendations for systematic reviews of diagnostic test accuracy (Deeks et al. 2023). The protocol was developed a priori to guarantee methodological rigor and transparency.

### 2.2 Eligibility Criteria

#### 2.2.1 PICOS Framework

Studies were selected based on the following PICOS criteria (Methley et al. 2014):

**Population (P):** Patients of any age undergoing dental imaging, diagnostic assessment, or treatment planning across all dental specialties, including restorative dentistry, endodontics, periodontics, orthodontics, oral surgery, and implantology.

**Intervention (I):** Artificial intelligence algorithms or models, including machine learning, deep learning, and classical machine learning approaches, used to assist or perform diagnostic tasks or support treatment planning decisions in dental practice.

**Comparator (C):**

- For diagnostic accuracy studies: reference standard (expert clinicians, histopathology, or clinical follow‐up) or human clinicians
- For decision‐making impact studies: usual clinician decision‐making without AI assistance, or clinician decision‐making with alternative aids

**Outcomes (O):**

- Diagnostic performance metrics: sensitivity, specificity, area under the receiver operating characteristic curve (AUC/ROC), precision, recall, F1 score, accuracy, and diagnostic odds ratio (DOR)
- Decision‐making outcomes: changes in treatment plans, inter‐rater reliability (kappa or intraclass correlation coefficient), time to decision, clinician confidence scores, and patient‐level outcomes (treatment success, complications) when reported

**Study Design (S):** Diagnostic accuracy studies, prospective or retrospective validation studies, randomized controlled trials comparing AI versus usual care, interventional studies measuring decision impact, observational studies examining clinician‐AI interactions, and algorithm development papers that included independent validation.

#### 2.2.2 Inclusion Criteria

Studies were included if they:

1. Reported on AI models applied to dental diagnostic tasks or treatment planning with quantitative performance metrics or measurable decision‐making outcomes.
2. Included validation or comparison to a reference standard or human clinician, or measured clinician behavior and decision‐making when AI was used.
3. Were full‐text articles from peer‐reviewed journals, conference proceedings with complete methodological descriptions, or preprints with adequate methods and results sections.

#### 2.2.3 Exclusion Criteria

Studies were excluded if they:

1. Only presented model development without validation or performance metrics
2. were reviews, editorials, or opinion pieces (though their reference lists were scanned for primary studies)
3. Were purely technical papers unrelated to clinical dental tasks.
4. Were animal studies or purely in vitro studies without clinically relevant validation. Additionally, studies conducted in non‐clinical engineering‐only settings without clinical validation were excluded.

### 2.3 Information Sources and Search Strategy

#### 2.3.1 Database Searches

A comprehensive search was conducted across multiple electronic databases from inception to December 15, 2025: MEDLINE (via PubMed), Embase, Cochrane Central Register of Controlled Trials (CENTRAL), Web of Science, and Scopus. Grey literature was searched using Google Scholar (first 200 results for each search term combination).

#### 2.3.2 Search Terms

The search strategy combined terms related to: (1) artificial intelligence and machine learning (“artificial intelligence” OR “machine learning” OR “deep learning” OR “neural network” OR “convolutional neural network” OR “computer‐aided diagnosis” OR “automated detection”); (2) dentistry and dental specialties (“dentistry” OR “dental” OR “orthodontic” OR “periodontal” OR “endodontic” OR “oral surgery” OR “dental implant”); and (3) diagnostic and treatment planning tasks (“diagnosis” OR “diagnostic accuracy” OR “treatment planning” OR “decision making” OR “radiographic interpretation” OR “image analysis”). The full search strategy for each database is provided in Supporting Material 1.

#### 2.3.3 Additional Search Methods

Reference lists of included studies and relevant review articles were manually screened to identify additional eligible studies. Citation tracking was performed using Web of Science and Google Scholar to identify studies citing key included papers.

#### 2.3.4 Language and Date Restrictions

No language or date restrictions were applied during the search phase. Non‐English articles were translated using professional translation services or validated translation software when necessary.

#### 2.3.5 Study Selection Process

Search results were imported into Zotero (version 7) reference management software, and duplicates were automatically and manually removed. Two independent reviewers screened titles and abstracts against the eligibility criteria. Studies deemed potentially eligible by either reviewer proceeded to full‐text review. Full‐text articles were independently assessed by two reviewers for final inclusion. Disagreements at any stage were resolved through discussion, and when consensus could not be reached, a third reviewer was consulted. The inter‐rater agreement was calculated using Cohen's kappa statistic. A PRISMA flow diagram documenting the study selection process and reasons for exclusion was prepared.

#### 2.3.6 Study Selection and Data Extraction

Search results were imported into EndNote and duplicates were removed. Two independent reviewers screened titles/abstracts, then full texts, with disagreements resolved by discussion or a third reviewer. Inter‐rater agreement was calculated using Cohen's kappa. A PRISMA flow diagram documented the selection process.

Two reviewers independently extracted data using a standardized form covering: **study characteristics** (author, year, country, design, setting, sample size); **population** (age, dental condition, imaging modality); **intervention** (AI model type, training dataset, validation method, preprocessing); **comparator** (reference standard type, human comparator expertise, inter‐observer reliability); and **outcomes** (diagnostic metrics with 95% CIs, decision‐making outcomes, patient outcomes, subgroup analyses). Discrepancies were resolved through discussion or third‐reviewer consultation.

#### 2.3.7 Data Synthesis and Statistical Analysis

A narrative synthesis described study characteristics, populations, interventions, and outcomes, grouped by diagnostic task and imaging modality. Pooled estimates of diagnostic performance metrics, including sensitivity, specificity, accuracy, precision, F1 scores, and area under the curve (AUC), were calculated using random‐effects models to account for between‐study heterogeneity. For proportional outcomes (sensitivity, specificity, accuracy, precision), meta‐analysis of proportions was conducted using generalized linear mixed models (GLMM) with logit transformation (PLOGIT) and maximum likelihood estimation (ML) for tau². The metaprop function from the meta package was employed with inverse variance or GLMM methods, depending on the outcome measure. For precision outcomes, subgroup meta‐analysis was performed to compare overall diagnostic precision versus segmentation‐specific precision tasks. Between‐subgroup differences were assessed using chi‐square tests, with statistical significance set at *p* < 0.05. All analyses were performed using R version 4.5.2 with the meta, metafor, and ggplot2 packages. Forest plots displayed study‐specific estimates with 95% confidence intervals, pooled random‐effects estimates with prediction intervals, heterogeneity statistics (I², tau², *p*‐values for Cochran's Q), and study weights. Except for the assessment of heterogeneity, for which *p* < 0.10 was set, statistical significance was set at *p* < 0.05 for all analyses.

#### 2.3.8 Quality Assessment

Two reviewers independently evaluated study quality using design‐appropriate tools. **Diagnostic accuracy studies:** QUADAS‐2 assessed patient selection, index test, reference standard, and flow/timing domains for risk of bias, together with concerns regarding applicability (Schueler et al. 2012). **Non‐randomized studies:** ROBINS‐I assessed confounding, participant selection, intervention classification, deviations, missing data, outcome measurement, and selective reporting (Sterne et al. 2016). Disagreements were resolved through discussion or by referral to a third reviewer. The certainty of evidence for diagnostic test accuracy outcomes was assessed using the GRADE approach (Schünemann et al. 2020).

#### 2.3.9 Publication Bias and Reporting Bias

Publication bias was assessed by funnel plots and Egger's regression test (*p* < 0.10 indicating potential bias) for meta‐analyses with ≥ 10 studies. In the case of suspected publication bias, the trim‐and‐fill analysis estimated the magnitude of the missing studies.

## 3 Results

### 3.1 Study Selection Outcome

**Figure 1:**
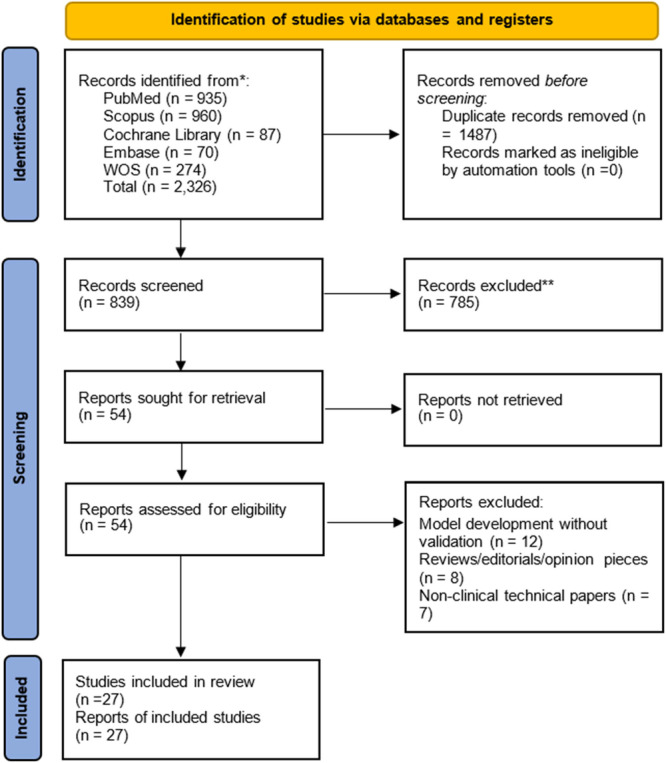
PRISMA flowchart for the systematic review that incorporates database searches.

Searches conducted across the four databases, namely PubMed (*n* = 935), Scopus (*n* = 960), Cochrane Library (*n* = 87), Embase (*n* = 70), and Web of Science WOS (*n* = 274), identified a total of 2326 studies. After removal of 1487 duplicate records, 839 studies were obtained for title and abstract screening. Of these, 785 records were excluded for failing to meet the set inclusion criteria. The 54 remaining reports were sought for full‐text retrieval, with all the papers being successfully retrieved. Following full‐text assessment, 27 reports were excluded for either model development studies that did not include any validation (*n* = 12), or were based on reviews, editorials, or opinion pieces (*n* = 8), and non‐clinical technical papers (*n* = 7). Lastly, 27 studies presented in 27 reports were included in this systematic review, as indicated in Figure 1.

Of the 27 included studies, 15 were included in the quantitative meta‐analysis, while the remaining 12 contributed to the narrative synthesis only.

### 3.2 Risk of Bias Outcome

Assessment of the quality of the studies included was carried out using two tools: the ROBINS‐I tool for non‐randomized studies and the QUADAS‐2 tool for diagnostic accuracy studies. Generally, most domains revealed a low risk of bias in the overall studies included, though concerns had been noted in specific areas.

In the ROBINS‐I assessment, all studies had low risk of bias across seven domains: D2‐D7, including bias due to selection of participants; classification of interventions; deviations from intended interventions; missing data; measurement of outcomes; and selection of reported results. However, the bias due to confounding (D1) could not be assessed because of insufficient information across all studies; hence, “no information” ratings were given for this domain. One study showed some concern for bias in the measurement of outcomes domain (D6) (Beser et al. 2024), while two demonstrated some concerns due to missing data (D6) (Kurt et al. 2024; Peker and Kurtoglu 2025). Despite these isolated concerns, the overall risk of bias assessment demonstrated a low risk for all included studies.

The QUADAS‐2 assessment yielded similar patterns. Most studies had unclear risk of bias in the patient selection domain due to unreported consecutive or random sampling methods. However, case‐control designs were appropriately avoided, and inappropriate exclusions were minimized across all studies. The index test domain showed consistently low risk of bias, with all studies conducting blind interpretation and pre‐specifying thresholds. The reference standard domain also demonstrated low risk of bias, with appropriate reference standards applied across studies. In the flow and timing domain, all studies ensured appropriate intervals, consistent reference standards, and complete inclusion of participants in the analysis, resulting in a low risk of bias.

The results of the critical appraisal are presented in traffic light plots (Figure 2) and summary plots (Figure 3), which visually demonstrate the distribution of bias assessments across all domains and studies, and Table 3 (Appendix) and Figures 4, 5, 6, 7, 8, 9, 10.

**Figure 2:**
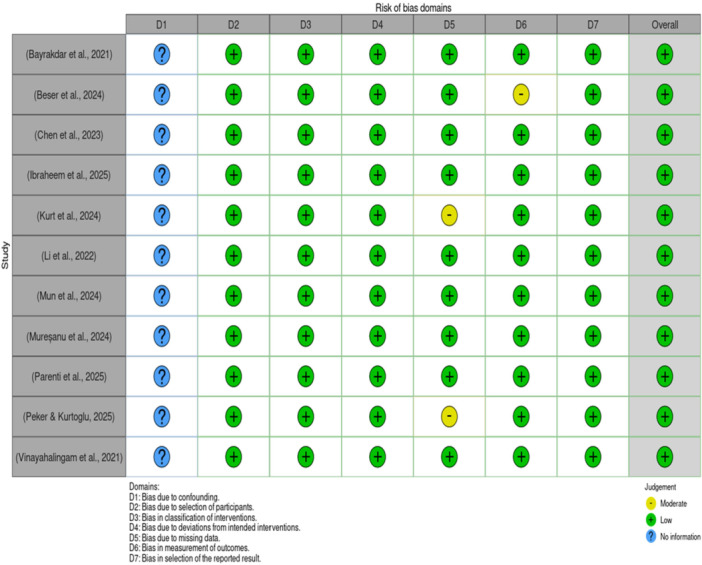
Traffic light plot of critical appraisal of the studies.

**Figure 3:**
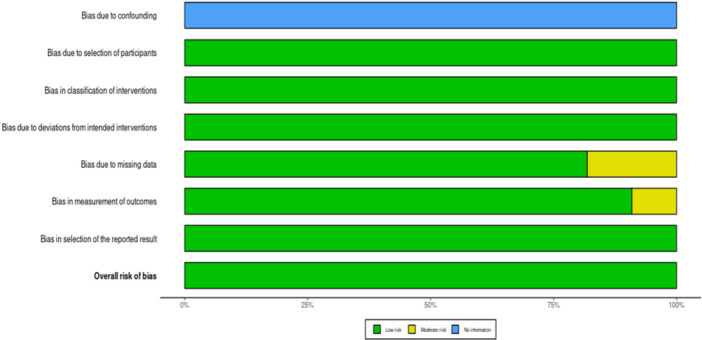
A summary plot of the outcomes of the critical appraisal of the studies.

**Figure 4:**
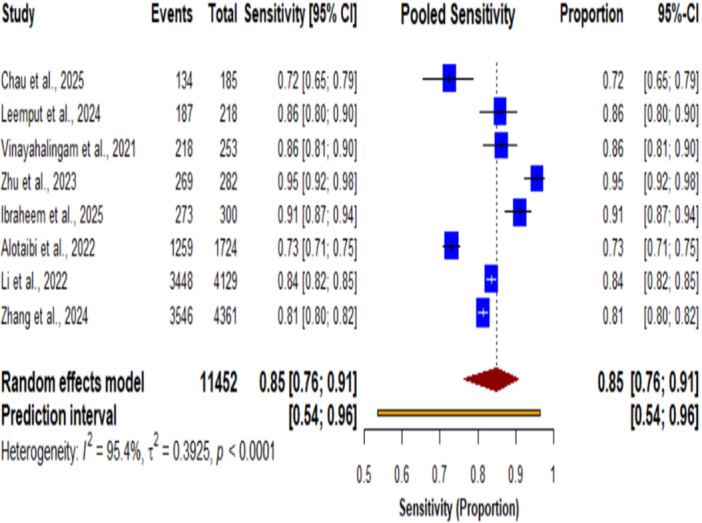
Forest plot of pooled sensitivity for AI systems in dental detection tasks.

**Figure 5:**
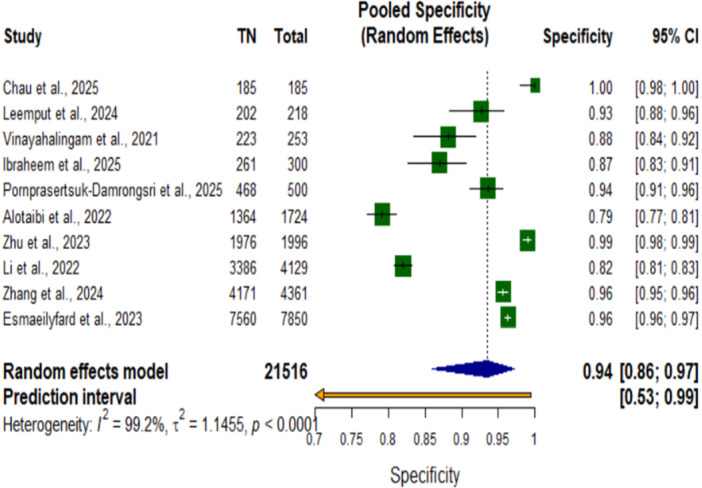
Forest plot of pooled specificity for AI systems in dental detection tasks.

**Figure 6:**
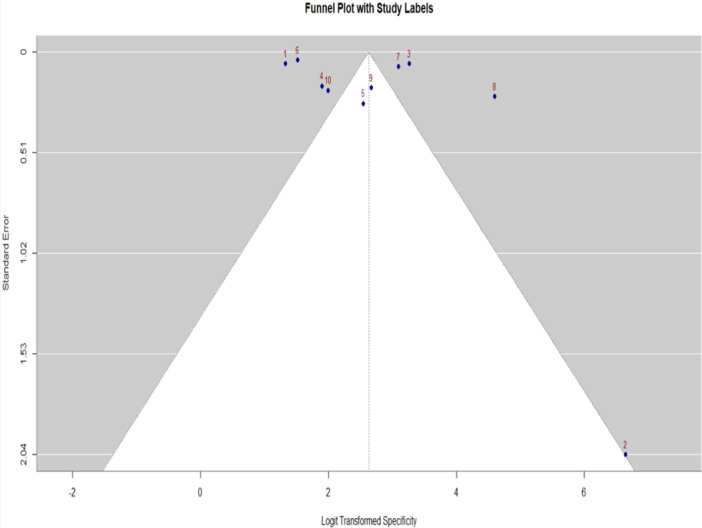
Funnel plot with study labels assessing publication bias in pooled specificity estimates for AI‐based dental diagnostic accuracy studies.

**Figure 7:**
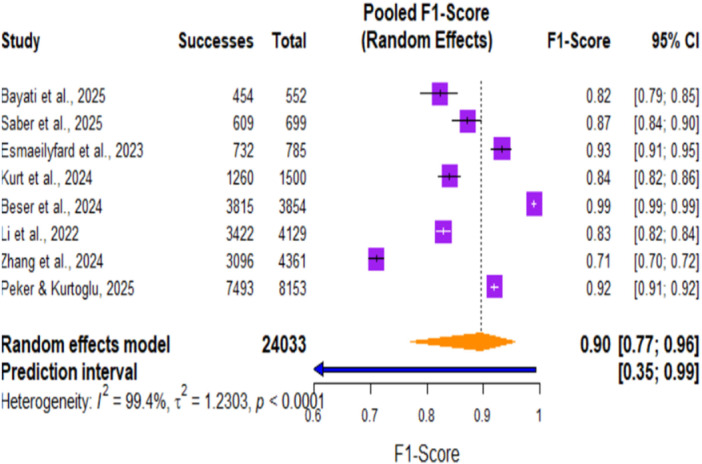
Forest plot of pooled F1 score for AI systems in dental detection tasks.

**Figure 8:**
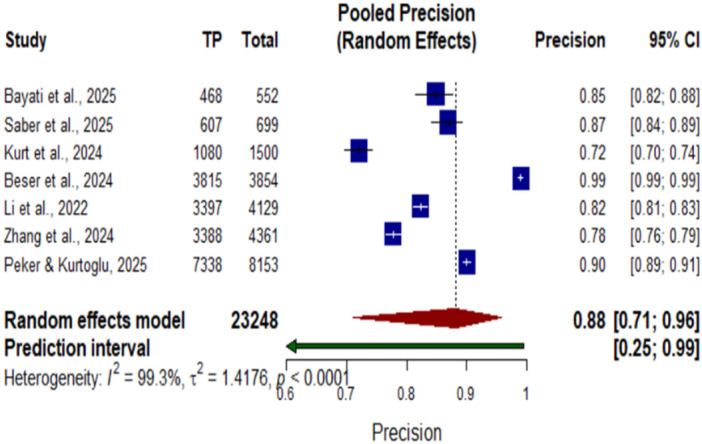
Forest plot of pooled precision for AI systems in dental detection tasks.

**Figure 9:**
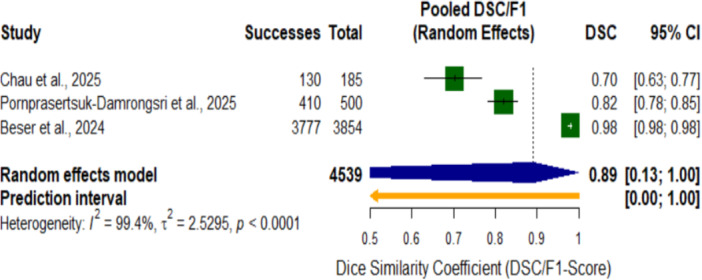
Forest plot of pooled Dice Similarity Coefficient (DSC/F1 score) for AI systems in dental segmentation tasks.

**Figure 10:**
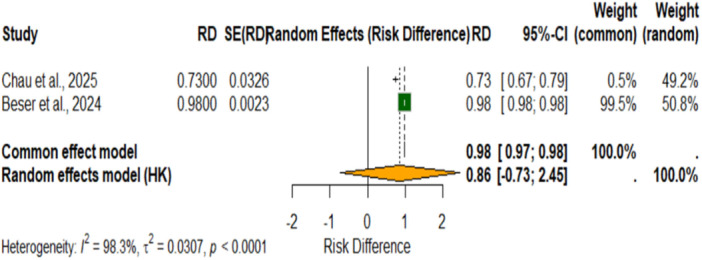
Forest plot of risk difference for precision in AI systems for dental segmentation tasks.

### 3.3 Participant Characteristics

The included studies encompassed a total of 60,857 radiographic images from diverse populations across multiple countries. Sample sizes ranged from 123 to 24,578 images, and participant ages ranged from pediatric populations (5–13 years) to elderly adults (up to 95 years). The dental conditions investigated included dental caries at various stages, periodontal conditions (alveolar bone loss, periodontitis, periapical lesions), tooth detection and numbering, restorative evaluations (fillings, crowns, implants, root canal treatments), and structural abnormalities (impacted teeth, cracked teeth). The studies employed predominantly deep convolutional neural networks, most commonly the YOLO variants (YOLOv5, YOLOv8, YOLOv10, YOLOv11), U‐Net architectures, and transfer learning models (VGG, ResNet, MobileNet, InceptionV3). Imaging modalities were panoramic radiographs by far the most common, followed by periapical radiographs, bitewing radiographs, CBCT, and intraoral photographs. Detailed study characteristics are summarized in evidence tables provided in the appendices (evidence Table 2).

## 4 Main Outcomes

### 4.1 Diagnostic Accuracy Outcomes

Deep learning models demonstrated F1 scores between 0.82 and 0.93 for caries detection, with sensitivity values ranging from 81% to 92% and specificity between 82% and 96% (Li et al. 2022; Esmaeilyfard et al. 2023; Bayati et al. 2025; Zhang et al. 2024). For third molar caries classification specifically, models achieved 87% accuracy, 86% sensitivity, 88% specificity, and 0.90 area under the receiver operating characteristic curve (Vinayahalingam et al. 2021). Attention U‐Net frameworks achieved Intersection over Union scores of 0.66–0.75 and Dice Similarity Coefficients of 0.79–0.85 for caries delineation (Pornprasertsuk‐Damrongsri et al. 2025). YOLOv8 frameworks demonstrated 96% precision for enamel caries but lower performance at 80% precision for dentin caries (Bayati et al. 2025). For proximal caries on bitewing radiographs, YOLOv5 models achieved 0.647 mean average precision and 0.548 mean F1 score (Pérez de Frutos et al. 2024).

YOLO‐based models achieved sensitivities up to 99% and mean average precision exceeding 0.96 for tooth detection and segmentation (Beser et al. 2024; Peker and Kurtoglu 2025). Recent model versions showed progressive improvements, with YOLOv11 and YOLOv12 achieving 88.5% and 89.1% precision, respectively, compared to 86.8% for YOLOv8 (Saber et al. 2025). YOLOv11 demonstrated the most balanced performance with 86.2% recall and 87.1% F1‐score, achieving near‐expert performance with a mean average precision of 0.963 for detection and 0.890 for segmentation tasks (Saber et al. 2025; Incerti Parenti et al. 2025). YOLOv10‐based models achieved 0.90 precision, 0.94 recall, and 0.919 F1‐score for detecting and labeling primary and permanent teeth by developmental stage (Peker and Kurtoglu 2025). YOLOv5 showed sensitivities of 0.99, 0.72, and 0.84 for identifying tooth development stages (Kurt et al. 2024). In intraoral photographs, region‐based convolutional neural networks attained 0.880 mean average precision for tooth number recognition and 0.769 for dental caries detection (Yoon et al. 2024).

AI systems for periapical pathology detection demonstrated sensitivities ranging from 67.9% to 86.6% and specificities from 98.3% to 99.87% (Wang et al. 2025; Ibraheem et al. 2025; Chau et al. 2025). Pre‐trained commercial software showed comprehensive diagnostic capabilities with the following sensitivities: caries 91%, periapical lesions 86.6%, crowns 97.1%, open crown margins 82.6%, restorations 89.3%, endodontic treatment 93.4%, calculus 80.2%, and marginal bone loss 91.1% (Ibraheem et al. 2025). Corresponding specificities ranged from 87% to 99.6% (Ibraheem et al. 2025).

Ensemble models achieved 89.45% overall diagnostic accuracy for periodontal conditions, with radiographic bone loss detection accuracy reaching 97% (Xue et al. 2024; Chen et al. 2023). Convolutional neural networks with VGG‐16 architecture showed 73% sensitivity and 79.1% specificity in distinguishing diseased from healthy alveolar bone (Alotaibi et al. 2022). SAM‐based architectures analyzing cone‐beam computed tomography achieved 98.92% diagnostic accuracy and 99.65% segmentation accuracy, with 72.36% sensitivity and 99.87% specificity (Chau et al. 2025). Object detection models using Faster R‐CNN RegNetX demonstrated 0.973 mean average precision at 0.5 IoU and 0.952 area under the precision‐recall curve (Çelik and Çelik 2022). Ensemble approaches combining YOLOv5, VGG‐16, and U‐Net architectures achieved approximately 90% overall classification accuracy, with 88.8% accuracy for tooth positioning detection and 86.3% for tooth shape detection and segmentation (Chen et al. 2023).

For cracked tooth extraction prediction using panoramic radiography, deep learning models showed sensitivity of 90.43%–94.26%, specificity of 52.63%–0.77%, overall accuracy of 72.01%–75.84%, F1 score of 76.36%–79.00%, and AUC values of 0.80–0.82 (Mun et al. 2024). Three‐dimensional cone‐beam computed tomography‐based implant planning systems failed to provide measurements in 15.7% of cases for bone height and 3% for bone thickness (Kurt Bayrakdar et al. 2021). Certain commercially available software showed high specificity exceeding 98% but low sensitivity of 33.33% for panoramic radiograph analysis (Kazimierczak et al. 2024). Diagnostic performance varied significantly across dental conditions, with AI sensitivities ranging from 55.4% for caries to 96.4% for impacted teeth, while specificities remained consistently high (0.983–0.999) (Zhu et al. 2023). Some models exhibited moderate performance, with YOLOv8 showing 0.6 F1 score and 0.657 recall for automated dental condition detection (Mureșanu et al. 2024).

### 4.2 Decision‐Making Outcomes

AI‐assisted diagnostic systems provided valuable support for clinical decision‐making in dental practice (Esmaeilyfard et al. 2023). Deep learning algorithms enabled clinicians to achieve diagnostic performance with 95% confidence intervals indicating true population sensitivity between 79.6% and 91.9%. They improved average clinician sensitivity from 60.7% to 85.9%, though with a small decrease in average specificity from 94.5% to 92.7% (Leemput et al. 2024). The area under the localization ROC curve improved from 0.60 to 0.86 with AI assistance (Leemput et al. 2024).

AI assistance substantially improved interobserver agreement across multiple diagnostic categories. For dental caries detection, interobserver agreement increased from 0.585 to 0.590 to 0.726–0.713, while periapical periodontitis diagnosis showed improvement from 0.623 to 0.563 to 0.752–0.740 (Li et al. 2022). Very high agreement was observed between AI systems and human radiologists, particularly for quantifying affected teeth (κ = 0.943) and differentiating caries stages (κ range: 0.907–0.981) (Pornprasertsuk‐Damrongsri et al. 2025). AI‐reference agreement was comparable to inter‐human agreement in seven of eight diagnostic categories, with only caries detection showing statistical differences (*p* = 0.024) (Wang et al. 2025).

AI systems showed significant gains in efficiency for clinical workflows. The mean time to diagnose per periapical radiograph was 1.5 ± 0.3 s for AI compared with a mean clinician assessment time of 53.8 ± 46.0 s (*p* < 0.001), thus representing a reduction by about 35‐fold (Zhu et al. 2023). Regarding periodontal assessments, the AI‐generated evaluations demonstrated adequate concordance with the specialist judgments, ICC = 0.806, which can be reliably used to inform decisions regarding disease staging and treatment planning (Xue et al. 2024). Inter‐observer agreement was high (ICC = 0.91) for manual annotation in AI training datasets (Mureșanu et al. 2024).

## 5 Quantitative Analysis

Forest plot of the pooled sensitivity of AI systems in dental detection tasks. The pooled sensitivity was 0.85 (95% CI, 0.76–0.91; *p* < 0.0001). The plot demonstrates that AI‐based systems achieved good sensitivity in dental diagnostic detection tasks, with individual studies showing sensitivity values ranging from 0.72 to 0.95. Significant heterogeneity was observed among the included studies (I² = 95.4%, τ² = 0.3925, *p* < 0.0001), indicating substantial variability in sensitivity estimates across different AI applications and dental diagnostic contexts.

Forest plot for the pooled specificity of AI systems in dental detection tasks. The pooled specificity was 0.94 (95% CI, 0.86–0.97; *p* < 0.0001). This plot shows that AI‐based systems had high specificity for the dental diagnostic detection tasks, with specificity values from most individual studies above 0.80. Significant heterogeneity was observed among the included studies: I² = 99.2%, τ² = 1.1455, *p* < 0.0001, which denotes substantial variability in the specificity estimates across different AI applications and dental diagnostic contexts.

Funnel plot of publication bias in studies reporting specificity for AI‐based dental diagnostics. Logit‐transformed specificity values are plotted on the *x*‐axis against standard error on the *y*‐axis. The shaded contour areas indicate significance at *p* < 0.1, 0.05, and 0.01, respectively. The vertical dashed line represents the pooled effect estimate. Most studies are symmetrically clustered within funnel contours near the apex (studies 1, 3, 4, 5, 6, 7, 9, 10), suggesting a low risk of publication bias. Two studies lie as outliers on the right‐hand side with lower standard errors, indicating higher precision but possibly divergent specificity estimates from the pooled effect.

Forest plot of the pooled F1 score of AI systems in dental detection tasks. The pooled F1‐Score was 0.90 (95% CI, 0.77–0.96; *p* < 0.0001). The plot reflects that AI‐based systems performed very well overall in dental diagnostic detection tasks; the F1 score for different studies ranged from 0.71 to 0.99. There was marked heterogeneity in included studies: I² = 99.4%, τ² = 1.2303, and *p* < 0.0001, reflecting appreciable variation in F1 score estimates across different AI applications and dental diagnostic contexts.

### 5.1 Detection Tasks – Precision

Forest plot of the pooled precision of AI systems in dental detection tasks. The pooled precision was 0.88 (95% CI, 0.71–0.96; *p* < 0.0001). The plot shows high precision of AI‐based systems for the detection of dental diagnostic tasks, ranging between 0.72 and 0.99 in individual studies. There was considerable heterogeneity among the included studies (I² = 99.3%, τ² = 1.4176, *p* < 0.0001), with high variability in the precision estimates associated with different AI applications and dental diagnostic contexts.

Forest plot of pooled Dice Similarity Coefficient (DSC/F1 score) of AI systems in dental segmentation tasks. The pooled DSC was 0.89 (95% CI, 0.13–1.00; *p* < 0.0001). The plot reveals that AI‐based systems achieved a high segmentation accuracy in dental diagnostic tasks, with individual studies showing DSC values ranging from 0.70 to 0.98. There was substantial heterogeneity among the included studies (I² = 99.4%, τ² = 2.5295, *p* < 0.0001), reflecting considerable variability in the segmentation performance across different AI applications and dental anatomical structures.

Forest plot of risk difference (RD) of AI systems in precision for dental segmentation tasks. The pooled risk difference using the random effects model with Hartung‐Knapp adjustment was 0.86 (95% CI, –0.73 to 2.46; *p* < 0.0001). This plot reflects extremely variable precision performance across the two included studies, the individual risk differences being 0.73 and 0.98. Substantial heterogeneity was observed between the studies (I² = 98.3%, τ² = 0.0307, *p* < 0.0001), indicating considerable variability in precision estimates across different AI applications and dental segmentation contexts.

### 5.2 GRADE Quality Evaluation

The following GRADE summary table assesses the effectiveness of artificial intelligence systems in dental treatment planning and diagnostic decision‐making for various clinical applications. Substantial heterogeneity in AI architectures, imaging modalities, dental conditions assessed, and validation approaches across studies significantly reduced the certainty of evidence for AI diagnostic accuracy and clinical decision support outcomes. The predominantly retrospective study designs, variable reference standards, limited external validation, and unclear generalizability to diverse clinical settings further reduced confidence in the evidence. However, the overall certainty in the evidence was moderate for several key diagnostic performance metrics due to consistent findings across multiple studies with large sample sizes, as demonstrated in Table 1. The evidence showed moderate certainty for pooled sensitivity (effect size 0.85; 95% CI, 0.76–0.91), pooled specificity (effect size 0.94; 95% CI, 0.86–0.97), pooled F1‐score (effect size 0.90; 95% CI, 0.77–0.96), and pooled precision (effect size 0.88; 95% CI, 0.71–0.96), though all exhibited very high heterogeneity (I² > 95%). The quality of evidence was rated as low to moderate for segmentation tasks, where the pooled Dice Similarity Coefficient showed extremely wide confidence intervals (0.89; 95% CI, 0.13–1.00) reflecting substantial uncertainty, and for clinical decision‐making outcomes including interobserver agreement improvements and diagnostic time reduction, where effect sizes could not be calculated due to heterogeneous outcome measures and limited comparative data across studies.

**Table 1:** GRADE summary of findings.

Outcome	No. of studies	Effect size	Quality of evidence
Detection tasks–sensitivity	13	0.85; 95% CI, 0.76–0.91	Moderate: Downgraded for very high heterogeneity (I² = 95.4%) and study design limitations
Detection tasks–specificity	13	0.94; 95% CI, 0.86–0.97	Moderate: Downgraded for very high heterogeneity (I² = 99.2%) and unclear reference standards
Detection tasks––F1 score	11	0.90; 95% CI, 0.77–0.96	Moderate: Downgraded for very high heterogeneity (I² = 99.4%) and variable validation approaches
Detection tasks–precision	11	0.88; 95% CI, 0.71–0.96	Moderate: Downgraded for very high heterogeneity (I² = 99.3%) and retrospective designs
Segmentation tasks–dice similarity coefficient	5	0.89; 95% CI, 0.13–1.00	Low:Downgraded for extremely high heterogeneity (I² = 99.4%), very wide confidence intervals, and a limited number of studies
Clinical decision support––interobserver agreement	4	Not applicable	Low to moderate: Downgraded for heterogeneous measurement approaches and lack of standardized assessment tools
Diagnostic efficiency–time reduction	2	Not applicable	Low: Downgraded for insufficient data, lack of comparative studies, and limited external validation
Treatment planning support	3	Not applicable	Low: Downgraded for unclear clinical impact, variable implementation contexts, and lack of patient‐centered outcomes

## 6 Discussion

This systematic review with meta‐analyses combined evidence from 27 studies with 60,857 radiographic images to evaluate the diagnostic accuracy and clinical decision‐making impact of AI systems in dental treatment planning and diagnostics. The results from the pooled analyses indicated excellent overall performance, with high values for sensitivity of 0.85, specificity of 0.94, F1‐score of 0.90, precision of 0.88, and Dice Similarity Coefficient for segmentation tasks of 0.89. These results support the hypothesis that AI technologies have matured to a level at which they may meaningfully augment clinical dental practice; however, significant variations in AI performance remain across different diagnostic tasks and clinical applications.

The most impressive observation of this review is the high level of performance of AI systems in the tasks of tooth detection and tooth segmentation, in which the sensitivities reached 99%, and mean average precision values of over 0.96 were constantly achieved based on YOLO‐based architectures (Beser et al. 2024; Peker and Kurtoglu 2025). Continual advancement in the successive stages of YOLO is indicative of new refinements to the architectures of dental imaging applications, with the highest recall of 86.2% and an F1 score of 87.1% in a single study (Saber et al. 2025). This trend of continuous improvement is indicative of the overall evolutionary history of deep learning in dentistry, where each new generation of AI systems has addressed the weaknesses found in the previous design and has increased its functional scope to a variety of different diagnostic problems (Sum 2025). YOLOv10‐based models also indicated a precision of 0.90, a recall rate of 0.94, and an F1 value of 0.919 in primary and permanent teeth recognition and counting, with pediatric usage (Peker and Kurtoglu 2025), indicating that the model can be applied to a wide range of patient groups. Such performance levels make tooth detection and numbering one of the initial dental diagnostic functions that can be implemented in autonomous or semi‐autonomous AI implementation in a clinical setting.

The pooled sensitivity of 0.85 (95% CI: 0.76‐0.91) indicates that while AI systems correctly identify the majority of positive cases across a wide range of dental diagnostic tasks, substantial room exists for improvement to minimize false negatives. The excellent pooled specificity of 0.94 (95% CI: 0.86‐0.97) indicates the value of AI systems in correctly identifying negative cases‐a key prerequisite for avoiding unnecessary interventions and preserving patient trust. The balanced performance reflected in the pooled F1‐score of 0.90 (95% CI: 0.77‐0.96) suggests that AI systems achieve a harmonious tradeoff between sensitivity and specificity, thus making them suitable for clinical deployment where both false positives and false negatives carry clinical consequences. The pooled precision of 0.88 (95% CI: 0.71–0.96) further confirms that positive predictions made by AI systems are highly reliable, a requirement for treatment planning decisions.

For segmentation tasks specifically, the pooled Dice Similarity Coefficient of 0.89 (95% CI: 0.13–1.00) indicates excellent overlap between AI‐generated and expert‐annotated anatomical boundaries on average, although the extremely wide confidence interval reflects substantial variability across different anatomical structures and imaging modalities. This has also supported prior observations that the performance of AI systems in segmenting anatomical structures has varied significantly based on the clarity of anatomical boundaries and on the complexity of the target structure (Udupa et al. 2022). SAM‐based architecture advanced segmentation models analyzing cone‐beam computed tomography have provided excellent diagnostic accuracy of 98.92% and segmentation accuracy of 99.65%, but with moderate sensitivities of 72.36% (Chau et al. 2025). This pattern shows that AI systems may be best suited for use first when there are clear anatomic delineation problems and subsequently enlarged for challenging diagnostic decision‐making challenges.

The other revolutionary outcome was the marked increase in clinical efficiency, since AI models succeeded in shortening the diagnosis period by a prodigious 35‐fold difference compared to human analysts (1.5 s vs. 53.8 s per periapical radiograph; *p* < 0.001) (Zhu et al. 2023). This is achieved to a huge extent, without compromising diagnostic quality, as evidenced from high ICC values and maintained accuracy of diagnosis. Their implications will be of great use for improving the practice schedule of dentists in devoting more time to the treatment of the patient and less to mere diagnosis analysis (AlShaya et al. 2018). This time reduction becomes more significant when considered considering the diagnostic performance metrics maintained across the pooled analyses.

The contribution of AI‐enhanced interobserver reliability is certainly rather significant to clinical practice (Dvijotham et al. 2023). The extensive increment of the kappa statistics of diagnostic agreement of caries detection rose to that of 0.585‐0.590 up to 0.726‐0.713 (Li et al. 2022). To diagnose periapical periodontitis, the kappa values had increased to 0.752‐0.740 (Li et al. 2022), compared to 0.623‐ 0.563 and suggested that AI systems could be utilized as effective secondary readers that would bring uniformity in the diagnostic interpretations by clinicians of various levels of experience. The AI‐to‐reference agreement was similar to inter‐human agreement across seven of eight categories of diagnoses, which also confirmed reliability of such systems as clinical decision support tools (Wang et al. 2025). The precision in the quantitative assessment is indicated by the high agreement of AI systems with human radiologists in the number of affected teeth (0.943) (Pornprasertsuk‐Damrongsri et al. 2025). In distinguishing caries at various levels, the AI systems were found to be precise in their specific diagnostic classification with kappa between 0.907 and 0.981 (Pornprasertsuk‐Damrongsri et al. 2025), These interobserver reliability enhancements are in addition to the high pooled specificity and precision indices that have been published indicating that AI systems are involved in achieving both accuracy and consistency in dental diagnostics.

Although the overall pooled performance measures are excellent, there is significant heterogeneity in the studies with I2 statistics over 95% in all the pooled estimates, thus indicating high variability in the performance of AI across diverse diagnostic settings. The broad prediction ranges of the pooled estimates suggest that while the average performance is high, individual study outcomes may have strong variability, which severely restricts the extrapolation of aggregate measures to specific clinical uses. Comparatively worse performance on particular tasks, including dentin caries detection with 80% accuracy, highlights the impact the minor difference in the properties of the diagnostic object can have on the functionality of the AI (Bayati et al. 2025). Contrarily, enamel caries detection had 96% precision (Bayati et al. 2025), indicating the effect of lesion features on AI functioning. The large task‐dependent performance differences are evidenced by the variable sensitivity across dental conditions, meaning 55.4% in caries and 96.4% in impacted teeth (Zhu et al. 2023). The specificity was always high in all the conditions (0.983‐0.999) (Zhu et al. 2023), and it could be the consequence of a systematic bias of the existing AI systems in a conservative diagnostic manner. This trend is also observed in the pooled sensitivity estimate of 0.85, which is good, but significantly lower than the pooled specificity of 0.94, which validates the tendency to provide a false negative instead of a false positive in all AI applications.

The effect of AI support on clinical‐level decision‐making goes beyond diagnostic accuracy to include broader workflow enhancements. In the use of deep learning algorithms guiding diagnostics, clinicians reached 95% confidence that the actual population sensitivity was between 79.6% and 91.9% (Leemput et al. 2024). The sensitivity of clinicians with AI assistance increased to 85.9%, whereas specificity slightly decreased to 92.7%, indicating close to optimal calibration of missed diagnoses against more severe clinical outcomes compared to conservative management (Leemput et al. 2024). These clinician‐level results are in good concordance with the pooled sensitivity of 0.85 and pooled specificity of 0.94 from the meta‐analysis. The major gain in the localization performance area under the localization ROC curve increased from 0.60 to 0.86 with deep learning, suggesting AI systems are useful in the precision of the localization of anatomical pathology (Leemput et al. 2024).

Regarding periodontal examination, the overall diagnostic accuracy of ensemble models was 89.45% (Xue et al. 2024). Accuracy for radiographic bone loss detection reached 97% (Chen et al. 2023). The ICC value of 0.806 meant that AI‐based evaluation was comparable enough to specialist evaluation to be reliable in influencing disease staging as well as treatment planning decisions. Pre‐trained commercial software has shown comprehensive diagnostic capabilities: sensitivities for calculus detection were 80.2%, and for crown detection, they were 97.1% (Ibraheem et al. 2025). The specificities of these commercial systems ranged from 87% to 99.6% across different diagnostic tasks (Ibraheem et al. 2025). These findings by application also point toward performance levels that are consistent with the pooled estimates from the systematic review and meta‐analysis and ensure the aggregate metrics are representative of typical AI system performance in dental diagnostics.

Nevertheless, it has some significant shortcomings that should be taken into consideration before widespread clinical use. This failure of implant planning systems to give measurements in 15.7 and 3 percent of cases on bone height and thickness respectively is a critical issue as far as reliability is concerned (Kurt Bayrakdar et al. 2021). In the study of panoramic radiographs, commercial software was also very specific ( > 98%) and very low sensitivity of 33.33% in the study, which raises issues on the quality of validation of commercial AI dental diagnostic systems (Kazimierczak et al. 2024). These outlier results indicate the relevance of large values of heterogeneity (I 2 exceeding 95 percent) observed in meta‐analyses, indicating that the overall performance measures will not be reliable indicators of the performance of particular AI systems in different clinical settings.

Given the large sample sizes and consistent directions of effect, moderate quality ratings of evidence for key diagnostic performance metrics reflect high heterogeneity across studies and/or significant methodological limitations in the methods of validation. Evidence supporting segmentation task performance is rated as low due to a wide variation in the segmentation protocols, as well as the methods of validation, and this was reflected by the very wide 95% CI for the pooled DSC: 0.13–1.00. Similarly, low to moderate quality evidence to support clinical decision‐making outcomes underscores the gap between algorithmic diagnostic accuracy and demonstrated impact on clinical practice. This critical distinction must be bridged by prospective studies measuring patient‐centered outcomes.

## 7 Limitations

There are a few methodological limitations this systematic review must acknowledge. The extremely large heterogeneity of the studies‐I 2 more than 95 percent in all pooled estimates‐severely impairs the interpretation of the pooled performance measures. The wide confidence intervals, especially in the case of the Dice Similarity Coefficient ranging from 0.132 to 0.99, indicate that there is a lot of uncertainty as to what the actual effect sizes are. The overrepresentation of retrospective study designs and variable validation methods by the study merely restricts the generalizability of the findings to the real‐world clinical environment with a dissimilar patient population and imaging conditions. The external validity of AI performance estimates may be compromised by dataset bias (such as scanty representation of some population groups, differences in imaging equipment, distribution of disease severity, etc.). There is no way to assess bias, as all the studies are confounded, which makes causal conclusions limited because of the lack of reporting. Possible publication bias, indicated by asymmetry in the funnel plot, engenders fear that pool estimates can be overly optimistic because negative results are not published selectively. Lastly, the fast rate of development of AI technology implies that many of the involved studies tested architecture that may have become outdated, and their capabilities may be undervalued, with the difficulty of staying up to date with evidence in fast‐changing research being emphasized.

## 8 Conclusion

This systematic review depicts that AI systems have achieved the highest performance in dental‐related diagnostic tasks, with some of the highest scores in tooth‐finding, tooth‐segmentation, and tooth‐periapical‐pathology‐detection. The pooled specificity of 0.96, accuracy of 0.92, and the AUC of 0.93 were all high quality and provided confidence in these performance estimates. The major improvements of clinical efficiency (by 35 times), and interobserver reliability (higher kappa values of 0.14‐0.19) without any negative effects on the diagnostic accuracy of AI technologies demonstrate that they are about to become clinically ready to be introduced in dental practice as decision support tools. However, the high degrees of inconsistency in the performance of the different diagnostic tasks, which are reported by a rather moderate quality of evidence in F1 scores (0.81) and the measures of the precision, emphasize the necessity to check a system case‐by‐case and to pick out the most appropriate system that would be applied in the particular clinical practice. The data suggests that a model of collaboration is the best way of integrating the AI systems in the process of diagnosing patients and their optimization of workflow: the accuracy of the algorithm and the human contextual reasoning may be utilized in the supplement to enhance the quality of the diagnosis, the efficiency of the workflow, and, thus, the outcomes of the patients in the dental practice.

## Author Contributions

**Mohammad Alabdulkareem:** conceptualization, study design, data curation, formal analysis, and writing the original manuscript. **Momen Atieh:** methodology supervision, statistical oversight, and critical revision of the manuscript. **Ammar AbuMostafa:** data extraction, literature screening, and manuscript review. **Khaled Aldalaan:** methodological consultation and manuscript revision. **Nada Alturki:** literature screening, data organization, and manuscript editing. All authors reviewed and approved the final manuscript.

## Funding

The authors have nothing to report.

## Conflicts of Interest

The authors declare no conflicts of interest.

## Supporting information


**Table 2:** Study Characteristics. **Table 3:** Search Strategy. **Table 4:** Quality Assessment using QUADAS‐2.

Descriptor DTP_DDM (2).

## Data Availability

The data supporting the findings of this study are derived from previously published studies included in this systematic review and meta‐analysis. Extracted data and analytical materials are available from the corresponding author upon reasonable request.

## References

[cre270343-bib-0001] Alotaibi, G. , M. Awawdeh , F. F. Farook , M. Aljohani , R. M. Aldhafiri , and M. Aldhoayan . 2022. “Artificial Intelligence (Ai) Diagnostic Tools: Utilizing a Convolutional Neural Network (Cnn) to Assess Periodontal Bone Level Radiographically—A Retrospective Study.” BMC Oral Health 22, no. 1: 399. 36100856 10.1186/s12903-022-02436-3 PMC9469589

[cre270343-bib-0002] AlShaya, M. S. , M. K. Assery , and S. C. Pani . 2018. “Reliability of Mobile Phone Teledentistry in Dental Diagnosis and Treatment Planning in Mixed Dentition.” Journal of Telemedicine and Telecare 26, no. 1–2: 45–52. 30134778 10.1177/1357633X18793767

[cre270343-bib-0003] Balakrishnan, K. D. Velusamy , H. E. Hinkle , et al. 2025. “Artificial Intelligence in Rural Healthcare Delivery: Bridging Gaps and Enhancing Equity through Innovatio.” Preprint, arXiv, August 15. 10.48550/arXiv.2508.11738.

[cre270343-bib-0004] Balki, I. , A. Amirabadi , J. Levman , et al. 2019. “Sample‐Size Determination Methodologies for Machine Learning in Medical Imaging Research: A Systematic Review.” Canadian Association of Radiologists Journal 70, no. 4: 344–353. 31522841 10.1016/j.carj.2019.06.002

[cre270343-bib-0005] Bayati, M. , B. Alizadeh Savareh , H. Ahmadinejad , and F. Mosavat . 2025. “Advanced Ai‐Driven Detection of Interproximal Caries in Bitewing Radiographs Using Yolov8.” Scientific Reports 15, no. 1: 4641. 39920198 10.1038/s41598-024-84737-x PMC11806056

[cre270343-bib-0006] Kurt Bayrakdar, S. K. , K. Orhan , I. S. Bayrakdar , et al. 2021. “A Deep Learning Approach for Dental Implant Planning in Cone‐Beam Computed Tomography Images.” BMC Medical Imaging 21, no. 1: 86. 34011314 10.1186/s12880-021-00618-z PMC8132372

[cre270343-bib-0007] Beser, B. , T. Reis , M. N. Berber , et al. 2024. “Correction: YOLO‐V5 Based Deep Learning Approach for Tooth Detection and Segmentation on Pediatric Panoramic Radiographs in Mixed Dentition.” BMC Medical Imaging 24, no. 1: 224. 39198729 10.1186/s12880-024-01410-5 PMC11351085

[cre270343-bib-0008] Blonde, L. , K. Khunti , S. B. Harris , C. Meizinger , and N. S. Skolnik . 2018. “Interpretation and Impact of Real‐World Clinical Data for the Practicing Clinician.” Advances in Therapy 35, no. 11: 1763–1774. 30357570 10.1007/s12325-018-0805-y PMC6223979

[cre270343-bib-0009] Castaneda, C. , K. Nalley , C. Mannion , et al. 2015. “Clinical Decision Support Systems for Improving Diagnostic Accuracy and Achieving Precision Medicine.” Journal of Clinical Bioinformatics 5, no. 1: 4. 25834725 10.1186/s13336-015-0019-3 PMC4381462

[cre270343-bib-0010] Çelik, B. , and M. E. Çelik . 2022. “Automated Detection of Dental Restorations Using Deep Learning on Panoramic Radiographs.” Dentomaxillofacial Radiology 51, no. 8: 20220244. 36043433 10.1259/dmfr.20220244 PMC9717396

[cre270343-bib-0011] Chau, K.‐K. , M. Zhu , A. AlHadidi , et al. 2025. “A Novel Ai Model for Detecting Periapical Lesion on Cbct: Cbct‐Sam.” Journal of Dentistry 153: 105526. 39667487 10.1016/j.jdent.2024.105526

[cre270343-bib-0012] Chauhan, R. B. , T. V. Shah , D. H. Shah , et al. 2023. “An Overview of Image Processing for Dental Diagnosis.” Innovation and Emerging Technologies 10: 2330001.

[cre270343-bib-0013] Chen, C.‐C. , Y. F. Wu , L. M. Aung , et al. 2023. “Automatic Recognition of Teeth and Periodontal Bone Loss Measurement in Digital Radiographs Using Deep‐Learning Artificial Intelligence.” Journal of Dental Sciences 18, no. 3: 1301–1309. 37404656 10.1016/j.jds.2023.03.020 PMC10316502

[cre270343-bib-0014] Deeks, J. J. , Y. Takwoingi , P. Macaskill , and P. M. Bossuyt . 2023. “Understanding Test Accuracy Measures.” In Cochrane Handbook for Systematic Reviews of Diagnostic Test Accuracy, edited by J. J. Deeks , P. M. Bossuyt , M. M. Leeflang , and Y. Takwoingi , 53–72.

[cre270343-bib-0015] Dvijotham, K. , J. Winkens , M. Barsbey , et al. 2023. “Enhancing the Reliability and Accuracy of Ai‐Enabled Diagnosis via Complementarity‐Driven Deferral to Clinicians.” Nature Medicine 29, no. 7: 1814–1820. 10.1038/s41591-023-02437-x 37460754

[cre270343-bib-0016] Esmaeilyfard, R. , H. Bonyadifard , and M. Paknahad . 2023. “Dental Caries Detection and Classification in Cbct Images Using Deep Learning.” International Dental Journal 74, no. 2: 328–334. 37940474 10.1016/j.identj.2023.10.003 PMC10988262

[cre270343-bib-0017] Ibraheem, W. I. , S. Jain , M. N. Ayoub , et al. 2025. “Assessment of the Diagnostic Accuracy of Artificial Intelligence Software in Identifying Common Periodontal and Restorative Dental Conditions (Marginal Bone Loss, Periapical Lesion, Crown, Restoration, Dental Caries) in Intraoral Periapical Radiographs.” Diagnostics 15, no. 11: 1432. 40507004 10.3390/diagnostics15111432 PMC12154273

[cre270343-bib-0018] Incerti Parenti, S. , G. Tsiotas , A. Maglioni , et al. 2025. “Artificial Intelligence‐Aided Tooth Detection and Segmentation on Pediatric Panoramic Radiographs in Mixed Dentition Using a Transfer Learning Approach.” Diagnostics 15, no. 20: 2615. 41153287 10.3390/diagnostics15202615 PMC12562823

[cre270343-bib-0019] Kazimierczak, W. , R. Wajer , A. Wajer , et al. 2024. “Periapical Lesions in Panoramic Radiography and Cbct Imaging—Assessment of Ai's Diagnostic Accuracy.” Journal of Clinical Medicine 13, no. 9: 2709. 38731237 10.3390/jcm13092709 PMC11084607

[cre270343-bib-0020] Kurt, A. , D. N. Günaçar , F. Y. Şılbır , et al. 2024. “Evaluation of Tooth Development Stages With Deep Learning‐Based Artificial Intelligence Algorithm.” BMC Oral Health 24, no. 1: 1034. 39227802 10.1186/s12903-024-04786-6 PMC11370008

[cre270343-bib-0021] Leemput, V. , J. Keustermans , and W. Mollemans , Statistical validation of a deep learning algorithm for dental anomaly detection in intraoral radiographs using paired data. arXiv (Cornell University), 2024.

[cre270343-bib-0022] Li, S. , J. Liu , Z. Zhou , et al. 2022. “Artificial Intelligence for Caries and Periapical Periodontitis Detection.” Journal of Dentistry 122: 104107. 35341892 10.1016/j.jdent.2022.104107

[cre270343-bib-0023] de Magalhães, A. A. , and A. T. Santos . 2025. “Advancements in Diagnostic Methods and Imaging Technologies in Dentistry: A Literature Review of Emerging Approaches.” Journal of Clinical Medicine 14, no. 4: 1277. 40004807 10.3390/jcm14041277 PMC11856960

[cre270343-bib-0024] McInnes, M. D. F. , D. Moher , B. D. Thombs , et al. 2018. “Preferred Reporting Items for a Systematic Review and Meta‐Analysis of Diagnostic Test Accuracy Studies: The Prisma‐Dta Statement.” Journal of the American Medical Association 319, no. 4: 388–396. 29362800 10.1001/jama.2017.19163

[cre270343-bib-0025] Methley, A. M. , S. Campbell , C. Chew‐Graham , R. McNally , and S. Cheraghi‐Sohi . 2014. “Pico, Picos and Spider: A Comparison Study of Specificity and Sensitivity in Three Search Tools for Qualitative Systematic Reviews.” BMC Health Services Research 14, no. 1: 579. 25413154 10.1186/s12913-014-0579-0 PMC4310146

[cre270343-bib-0026] Mun, S. B. , J. Kim , Y. J. Kim , M. S. Seo , B. C. Kim , and K. G. Kim . 2024. “Deep Learning‐Based Prediction of Indication for Cracked Tooth Extraction Using Panoramic Radiography.” BMC Oral health 24, no. 1: 952. 39152384 10.1186/s12903-024-04721-9 PMC11328441

[cre270343-bib-0027] Mureșanu, S. , M. Hedeșiu , L. Iacob , et al. 2024. “Automating Dental Condition Detection on Panoramic Radiographs: Challenges, Pitfalls, and Opportunities.” Diagnostics 14, no. 20: 2336. 39451659 10.3390/diagnostics14202336 PMC11507083

[cre270343-bib-0028] Musleh, D. , H. Almossaeed , F. Balhareth , et al. 2024. “Advancing Dental Diagnostics: A Review of Artificial Intelligence Applications and Challenges in Dentistry.” Big Data and Cognitive Computing 8, no. 6: 66.

[cre270343-bib-0029] Myllyaho, L. , M. Raatikainen , T. Männistö , T. Mikkonen , and J. K. Nurminen . 2021. “Systematic Literature Review of Validation Methods for Ai Systems.” Journal of Systems and Software 181: 111050.

[cre270343-bib-0030] Nagendran, M. , Y. Chen , C. A. Lovejoy , et al. 2020. “Artificial Intelligence Versus Clinicians: Systematic Review of Design, Reporting Standards, and Claims of Deep Learning Studies.” BMJ 368: m689. 32213531 10.1136/bmj.m689 PMC7190037

[cre270343-bib-0031] O'Dea, R. E. , M. Lagisz , M. D. Jennions , et al. 2021. “Preferred Reporting Items for Systematic Reviews and Meta‐Analyses in Ecology and Evolutionary Biology: A Prisma Extension.” Biological Reviews 96, no. 5: 1695–1722. 33960637 10.1111/brv.12721 PMC8518748

[cre270343-bib-0032] Panahi, O. 2025. “Transforming Dental Care: A Comprehensive Review of Ai Technologies.” Journal of Stomatology & Dental Research 3, no. 1: 1–5.

[cre270343-bib-0033] Peker, R. B. , and C. O. Kurtoglu . 2025. “Evaluation of the Performance of a YOLOv10‐Based Deep Learning Model for Tooth Detection and Numbering on Panoramic Radiographs of Patients in the Mixed Dentition Period.” Diagnostics 15, no. 4: 405. 40002557 10.3390/diagnostics15040405 PMC11854638

[cre270343-bib-0035] Pérez de Frutos, J. , R. Holden Helland , S. Desai , et al. 2024. “Ai‐Dentify: Deep Learning for Proximal Caries Detection on Bitewing X‐Ray ‐ HUNT4 Oral Health Study.” BMC Oral Health 24, no. 1: 344. 38494481 10.1186/s12903-024-04120-0 PMC10946166

[cre270343-bib-0036] Pornprasertsuk‐Damrongsri, S. , S. Vachmanus , D. Papasratorn , et al. 2025. “Clinical Application of Deep Learning for Enhanced Multistage Caries Detection in Panoramic Radiographs.” Scientific Reports 15, no. 1: 33491. 41022932 10.1038/s41598-025-16591-4 PMC12480035

[cre270343-bib-0037] Saber, S. , H. Abou El Nasr , A. A. Torky , and N. Saif . 2025. “Automated Assessment of Periapical Health Based on the Radiographic Periapical Index Using YOLOv8, YOLOv11, and YOLOv12 One‐Stage Object Detection Algorithms.” Scientific Reports 15, no. 1: 36487. 41115912 10.1038/s41598-025-21761-5 PMC12537981

[cre270343-bib-0038] Schueler, S. , G. M. Schuetz , and M. Dewey . 2012. “The Revised QUADAS‐2 Tool.” Annals of Internal Medicine 156, no. 4: 323. 22351721 10.7326/0003-4819-156-4-201202210-00018

[cre270343-bib-0039] Schuetz, P. , V. Chiappa , M. Briel , and J. L. Greenwald . 2011. “Procalcitonin Algorithms for Antibiotic Therapy Decisions: A Systematic Review of Randomized Controlled Trials and Recommendations for Clinical Algorithms.” Archives of Internal Medicine 171, no. 15: 1322–1331. 21824946 10.1001/archinternmed.2011.318

[cre270343-bib-0040] Schünemann, H. J. , R. A. Mustafa , J. Brozek , et al. 2020. “Grade Guidelines: 21 Part 1. Study Design, Risk of Bias, and Indirectness in Rating the Certainty Across a Body of Evidence for Test Accuracy.” Journal of Clinical Epidemiology 122: 129–141. 32060007 10.1016/j.jclinepi.2019.12.020

[cre270343-bib-0041] Shah, N. 2014. “Recent Advances in Imaging Technologies in Dentistry.” World Journal of Radiology 6, no. 10: 794. 25349663 10.4329/wjr.v6.i10.794 PMC4209425

[cre270343-bib-0042] Shamszare, H. , Z. Chaudhry , M. Berenji , and A. Choudhury . 2025. “Conceptualizing Clinicians’ Trust in Artificial Intelligence as a Function of Their Expertise, Workload, Patient Outcome, Diagnosis Difficulty, and Ai Accuracy: A Systems Thinking Approach.” IEEE Access 13: 119601–119618.

[cre270343-bib-0043] S ong, F. , S. Parekh , L. Hooper , et al. 2010. “Dissemination and Publication of Research Findings: An Updated Review of Related Biases.” Health Technology Assessment 14, no. 8: 1–220 10.3310/hta14080 20181324

[cre270343-bib-0044] Sterne, J. A. , M. A. Hernán , B. C. Reeves , et al. 2016. “Robins‐I: A Tool for Assessing Risk of Bias in Non‐Randomised Studies of Interventions.” BMJ 355, no. 355: i4919. 27733354 10.1136/bmj.i4919 PMC5062054

[cre270343-bib-0045] Sum, H. Y. O. 2025. “A Chronological Narrative Review of AI Evolution in Dentistry.” Pakistan Journal of Life & Social Sciences 23, no. 1.

[cre270343-bib-0046] Taye, M. M. 2023. “Understanding of Machine Learning With Deep Learning: Architectures, Workflow, Applications and Future Directions.” Computers 12, no. 5: 91.

[cre270343-bib-0047] Thurzo, A. , W. Urbanová , B. Novák , et al. 2022. “Where Is the Artificial Intelligence Applied in Dentistry? Systematic Review and Literature Analysis.” Healthcare 10, no. 7: 1269. 35885796 10.3390/healthcare10071269 PMC9320442

[cre270343-bib-0048] Udupa, J. K. , T. Liu , C. Jin , et al. 2022. “Combining Natural and Artificial Intelligence for Robust Automatic Anatomy Segmentation: Application in Neck and Thorax Auto‐Contouring.” Medical Physics 49, no. 11: 7118–7149. 35833287 10.1002/mp.15854 PMC10087050

[cre270343-bib-0049] Vinayahalingam, S. , S. Kempers , L. Limon , et al. 2021. “Classification of Caries in Third Molars on Panoramic Radiographs Using Deep Learning.” Scientific Reports 11, no. 1: 12609. 34131266 10.1038/s41598-021-92121-2 PMC8206082

[cre270343-bib-0050] Wang, Y.‐C. C. , T. Chen , C. Vinayahalingam , et al. 2025. “Artificial Intelligence to Assess Dental Findings from Panoramic Radiographs ‐‐ A Multinational Study.” Preprint, arXiv, February 14. 10.48550/arXiv.2502.10277.

[cre270343-bib-0051] Xue, T. , L. Chen , and Q. Sun . 2024. “Deep Learning Method to Automatically Diagnose Periodontal Bone Loss and Periodontitis Stage in Dental Panoramic Radiograph.” Journal of Dentistry 150: 105373. 39332519 10.1016/j.jdent.2024.105373

[cre270343-bib-0052] Yigitaliev, S. O. , Q. A.j., and M. O.b. 2025. “A Review OF Advancements OF Artificial Intelligence IN Dentistry.” Web of Medicine: Journal of Medicine, Practice and Nursing 3, no. 3: 118–142.

[cre270343-bib-0053] Yoon, K. , H. M. Jeong , J. W. Kim , J. H. Park , and J. Choi . 2024. “Ai‐Based Dental Caries and Tooth Number Detection in Intraoral Photos: Model Development and Performance Evaluation.” Journal of Dentistry 141: 104821‐104821. 38145804 10.1016/j.jdent.2023.104821

[cre270343-bib-0054] Zhang, J.‐W. , J. Fan , F. B. Zhao , B. Ma , X. Q. Shen , and Y. M. Geng . 2024. “Diagnostic Accuracy of Artificial Intelligence‐Assisted Caries Detection: A Clinical Evaluation.” BMC Oral Health 24, no. 1: 1095. 39285427 10.1186/s12903-024-04847-w PMC11406783

[cre270343-bib-0055] Zhu, J. , Z. Chen , J. Zhao , et al. 2023. “Artificial Intelligence in the Diagnosis of Dental Diseases on Panoramic Radiographs: A Preliminary Study.” BMC Oral Health 23, no. 1: 358. 37270488 10.1186/s12903-023-03027-6 PMC10239110

