# Supplementary material for: Artificial Intelligence in Dental Treatment Planning and Diagnostic Decision‐Making: A Systematic Review and Meta‐Analysis
Source: Clin Exp Dent Res. 2026 Mar 31;12(2):e70343. doi: 10.1002/cre2.70343 (PMC13140480; doi:10.1002/cre2.70343)
Supplement: Supplementary file 1 — Table 2: Study Characteristics. Table 3: Search Strategy. Table 4: Quality Assessment using QUADAS‐2. [file CRE2-12-e70343-s001.docx]

**Appendix**

Table 2: Study Characteristics

| **Study (Author, Year)** | **Country / Setting** | **Study Design** | **Population / Sample Size** | **Age Range / Mean Age** | **Dental Condition / Pathology** | **AI Method / Algorithm Used** | **Imaging / Data Type** | **Comparator / Control** | **Intervention / AI Application** | **Outcome Measures** | **Key Findings / Performance Metrics** |
| --- | --- | --- | --- | --- | --- | --- | --- | --- | --- | --- | --- |
| (Alotaibi et al., 2022) | Saudi Arabia | Retrospective observational diagnostic AI study | 1,724 periapical radiographs from 1,610 patients | Adults; >12 years | Alveolar bone loss and periodontitis severity | Deep CNN (VGG-16), Transfer Learning, TensorFlow/Keras | Intraoral periapical radiographs (PNG, grayscale, 150×150 px) | Consensus of 3 calibrated examiners (including periodontist) | AI-based detection and staging of alveolar bone loss | Accuracy, precision, recall, F1-score, confusion matrix, MCC, Cohen Kappa, ROC, sensitivity, specificity | Binary classification achieved 73.04% accuracy with 73% sensitivity and 79.1% specificity, while multiclass accuracy was 59.42% with best performance for normal bone detection and poorest for mild loss detection. |
| (Bayati et al., 2025) | Iran | Retrospective deep learning diagnostic study | 552 bitewing radiographs, expanded to 1,506 images | Permanent teeth only | Interproximal dental caries (enamel and dentin) | YOLOv8 with segmentation; 200 epochs with augmentation | Bitewing radiographs (JPEG); PSP digital plates | Expert radiologist, assistant, third reviewer for discrepancies | Automated caries detection and segmentation using YOLOv8 | Precision, recall, F1 score, false negative rate, Dice coefficient, precision-confidence curves, recall-confidence curves | YOLOv8 achieved 96.03% precision for enamel caries and 80.06% for dentin caries, with overall precision 84.83%, recall 79.77%, F1 82.22%, and Dice coefficient 0.7949, significantly improving early lesion detection. |
| (Bayrakdar et al., 2021) | Turkey | Retrospective observational study | 75 CBCT images; 508 implant-site measurements | Not reported | Missing teeth requiring implant planning; bone evaluation; canal, sinus, fossae detection | Diagnocat deep CNN using modified 3D U-Net | CBCT; Promax 3D Mid scanner (Planmeca) | Manual assessment by oral and maxillofacial radiologist using InvivoDental 6.0 | Automatic AI implant planning: segmentation and bone measurement | Differences AI vs. manual (Wilcoxon, Bland–Altman); structure detection correctness; reliability (ICC, TEM) | AI correctly identified 72.2% of canals, 66.4% of sinuses/fossae, and 95.3% of missing tooth regions with excellent examiner reliability but significant measurement differences in bone thickness across all regions. |
| (Beser et al., 2024) | Turkey | Retrospective observational (non-interventional) | 3854 panoramic radiographs of pediatric patients | 5–13 years | Mixed dentition including deciduous and permanent teeth; caries, restorations, anomalies | YOLO-v5x deep CNN | Panoramic radiographs (Planmeca Promax 2D) | Manual expert labelling by orthodontists, pediatric dentists, radiologists | Automatic detection, segmentation, and numbering of teeth using YOLO-v5x | Sensitivity, precision, F1-score, mAP@0.5; confusion matrix | Detection model achieved sensitivity 0.99, precision 0.99, F1-score 0.99, mAP-0.5 0.98; segmentation model achieved sensitivity 0.98, precision 0.98, F1-score 0.98, mAP-0.5 0.98 with near-perfect performance. |
| (Çelik & Çelik, 2022) | Turkey | Diagnostic model development and validation | 123 panoramic radiographs with 684 labeled objects | 18–65 years | Dental restorations, denture, implant | 10 deep learning detection models including R-CNN, Faster R-CNN, SSD, YOLOv3, RetinaNet with various backbones | Panoramic radiographs | Comparison among 10 AI models | Automatic detection and localization of restorations, dentures, implants | mAP, AR, Precision-Recall curve, AUC | mAP ranged 0.755–0.973 with best performance by Faster R-CNN RegnetX achieving mAP 0.973, AR 0.771, AUC 0.952, demonstrating high potential for clinical AI-assisted detection. |
| (Chau et al., 2025) | China | Diagnostic model development and validation | 185 CBCT scans with 185 confirmed periapical lesions | Adults, not specified | Periapical lesions (various tooth locations) | CBCT-SAM, CBCT-SAM without PPR, Modified U-Net, PAL-Net | CBCT | Comparison among 4 AI models | Automatic identification and segmentation of periapical lesions using AI | Diagnostic accuracy, segmentation accuracy, sensitivity, specificity, precision, DSC | CBCT-SAM achieved diagnostic accuracy 98.92%, segmentation accuracy 99.65%, sensitivity 72.36%, specificity 99.87%, precision 0.73, DSC 0.70, providing expert-level assistance for early detection. |
| (Chen et al., 2023) | Taiwan | Observational (retrospective; STROBE-compliant) | 270 patients; 8000 periapical radiographs; 27,964 teeth | Mean 59.81 ± 10.53 years | Tooth position; tooth shape; periodontal bone level; radiographic bone loss | YOLOv5, VGG-16, U-Net, Mask R-CNN, Detectron2 (FPN), Deep Learning Hybrid | Periapical radiographs | Clinicians' assessments as reference | AI-based detection of tooth position, shape, bone level, and RBL | Accuracy, AP (AP50, AP75), segmentation accuracy | Tooth position 88.8%, tooth shape 86.3%, bone level 92.61%, RBL ~97% accuracy; AI outperformed dentists demonstrating strong potential for clinical periodontal diagnostics. |
| (Esmaeilyfard et al., 2023) | Iran | Diagnostic accuracy study | 785 CBCT molar cases; augmented to 7850 images | Not reported | Dental caries (presence, type, depth) | Multiple-input Deep CNN in PyTorch; 3-input CNN (axial, sagittal, coronal) | CBCT images (axial, sagittal, coronal slices), 96×160 px | Consensus of 2 experienced oral & maxillofacial radiologists | AI-based automatic detection and classification of caries | Accuracy, Sensitivity, Specificity, F1-score | Detection accuracy 95.3% (carious), 94.8% (noncarious), sensitivity 92.1%, specificity 96.3%, F1-score 93.2%; type classification accuracy 91.6–97.2%; extension accuracy 89.7–96.2%, demonstrating high reliability. |
| (Frutos et al., 2024) | Norway | Diagnostic accuracy study; 5-fold cross-validation | 13,887 bitewing images annotated by 6 clinicians; 197 consensus test images | 19–94 years | Proximal dental caries (enamel, dentine, secondary, unknown grade) | RetinaNet (ResNet50), YOLOv5 (M), EfficientDet (D0, D1) with transfer learning | Bitewing radiographs from HUNT4 Study | 6 experienced dental clinicians; consensus test set | Automated detection and classification of proximal caries | mAP, AP per class, mF1, mFNR, bootstrap CI | YOLOv5 outperformed clinicians with mAP 0.647, mF1 0.548, mFNR 0.149 achieving highest AP and lowest FNR across all classes with strong potential for diagnostic support. |
| (Ibraheem et al., 2025) | Saudi Arabia | Retrospective study | 300 IOPARs representing 1030 teeth | Adults (>18 years) | Caries, periapical lesions, crowns, open margins, restoration, endodontic treatment, calculus, bone loss | Pre-trained CADe AI (Second Opinion® v1.1), computer vision and ML | IOPARs | 2 experienced oral radiologists | AI-assisted diagnosis by interns and specialists | Sensitivity, specificity, PPV, NPV, accuracy, Youden's index | AI sensitivity ranged 80.2–97.1%, specificity 87–99.6%; operators using AI showed improved accuracy, particularly interns with caries accuracy increase of 23.62% and calculus sensitivity increase of 57.14%. |
| (Kazimierczak et al., 2024) | Poland | Retrospective diagnostic accuracy study | 49 patients, 1223 teeth | Mean 41 (range 12–70) | Periapical lesions | Diagnocat AI (cloud-based, automatic detection) | OPG and CBCT images | Consensus of 3 experienced clinicians (CBCT-based) | AI-assisted PL detection on OPG and CBCT | Sensitivity, specificity, accuracy, PPV, NPV, F1 score | AI sensitivity: OPG 33.33% (F1 32.73%), CBCT 77.78% (F1 84.00%); specificity >98% for both; demonstrated high CBCT accuracy but low OPG sensitivity with false negatives from metal artifacts. |
| (Kurt et al., 2024) | Turkey | Retrospective study | 1500 panoramic radiographs | 5–14 years, mean 8.43 ± 2.2 | Tooth development stages | YOLOv5 (CNN-based object detection) | Panoramic radiographs | Labeled ground truth according to Demirjian Method | AI-assisted automatic detection of tooth calcification/development stages | TP, FP, FN, sensitivity, precision, F1-score | Test set: TP=828, FP=308, FN=1; sensitivity 0.99, precision 0.72, F1-score 0.84; successfully detected 46 tooth development stage classes with high chronological evaluation accuracy. |
| (Leemput et al., 2024) | United States | Clinical validation study | 218 IOR images (validation); 3202 training; 936 tuning | Not specified | Caries, apical lesions, root canal defects, marginal defects, bone loss, calculus | CNN based on U-Net with pre-trained VGG19 | Periapical and bitewing IORs (digital sensors and PSP plates) | Dentists evaluating same IORs without AI | AI anomaly detection providing probabilistic segmentation and bounding boxes | Sensitivity, specificity, LROC, AUC, McNemar's test, binomial test | Sensitivity increased from 60.7% to 85.9%, AUC improved from 0.60 to 0.86 with all improvements statistically significant; largest gains for marginal and root canal defects. |
| (Li et al., 2022) | China | Retrospective experimental study | 4,129 periapical radiographs (3,829 training; 300 test) | Median 43 (range 20–79) | Dental caries, Periapical periodontitis | Modified ResNet-18 (two cascaded backbones), baseline VGG16 | Periapical radiographs | Junior dentists, VGG16 baseline | Automated detection of caries and periapical periodontitis | SEN, SPEC, PPV, NPV, F1-score, AUC, Fleiss κ | Caries: SEN 0.8350, SPEC 0.8200, F1 0.8288; Periodontitis: SEN 0.8200, SPEC 0.8400, F1 0.8283; outperformed VGG16 and improved junior dentist F1 and interobserver agreement substantially. |
| (Mun et al., 2024) | Republic of Korea | Retrospective non-interventional study | 418 teeth from 200 patients (209 normal, 209 cracked) | 21–89 years | Cracked tooth requiring extraction (VRF) | InceptionV3, ResNet50, EfficientNetB0 | Panoramic radiographs | Human diagnosis by endodontist | Automated prediction of cracked tooth extraction indications | Sensitivity, Specificity, Accuracy, F1 score, AUC-ROC | Sensitivity 90.43–94.26%, specificity 52.63–60.77%, accuracy 72.01–75.84%, F1 76.36–79.00%, AUC-ROC 0.80–0.82; ResNet50 showed highest AUC demonstrating statistically significant diagnostic performance. |
| (Mureșanu et al., 2024) | Romania | Retrospective, multi-center study | 1628 PRs (internal), 180 PRs (external validation) | Adult with permanent dentition | Periapical lesions, impacted teeth, root fragments, prosthetics, endodontics, caries, bone loss, orthodontic/surgical devices | YOLOv8 object detection | PRs (JPEG, resized 1024 × 1024 px) | Ground truth by 3 calibrated researchers | Automated detection of dental conditions and pre-radiotherapy risk assessment | Precision, Recall, F1 score, mAP@50, confusion matrix | Internal: F1=0.6, recall=0.657; External: F1=0.47, recall=0.451; precision/recall >0.8 for several conditions in training but performance declined during external validation indicating limited generalizability. |
| (Parenti et al., 2025) | Italy | Retrospective study | 250 pediatric PRs (25 test, 225 train+validation); 650 adult PRs for transfer learning | 6–13 years (mean 8.4 ± 1.8 in test) | Deciduous and permanent teeth, mixed dentition | YOLOv11 with hybrid pre-annotation and transfer learning | PRs (PNG, 2454 × 1304 px) | Expert manual annotation and FDI numbering system | Automated detection, classification, and segmentation of teeth during mixed dentition | Precision, recall, F1-score, mAP, confusion matrix | Detection: mAP0.5=0.963, macro F1=0.953, precision=0.946, recall=0.945; Segmentation: mAP0.5=0.890, F1=0.891; permanent teeth F1=0.977, deciduous F1=0.884 with misclassifications in overlapping deciduous teeth. |
| (Peker & Kurtoglu, 2025) | Turkey | Retrospective study | 200 pediatric PRs, 8153 teeth; 70% train, 15% validation, 15% test | 5–13 years | Mixed dentition: primary teeth and permanent tooth germs | YOLOv10 (Extra Large version) | PRs (PaX-Flex, 50–90 kV, 4–10 mA, 10.1 s) | Expert manual labeling with FDI numbering | Automated detection, numbering, and classification of primary and permanent teeth | Precision, recall, mAP50, mAP50-95, F1 score, confusion matrix | Weighted average: Precision=0.90, Recall=0.94, mAP50=0.968, mAP50-95=0.696, F1=0.919; tooth-specific F1 ranged 0.621–0.989 with most teeth >0.9 mAP50 and misclassification mainly for tooth 51. |
| (Pérez et al., 2023) | Norway | Observational with AI model training and evaluation | 4,913 participants (13,887 bitewing images) | 19–94 years (approx. mean 57) | Dental caries (enamel, dentine, secondary) | RetinaNet (ResNet50), YOLOv5 (M), EfficientDet (D0, D1) | Bitewing radiographs | 6 expert annotators, consensus test set of 197 images | AI-assisted caries detection using object detection models | mAP, mF1, mFNR | YOLOv5 highest: mAP 0.647, mF1 0.548, mFNR 0.149; best human: mAP 0.299, mF1 0.495, mFNR 0.164; AI generally performed similar to or better than experts. |
| (Pornprasertsuk-Damrongsri et al., 2025) | Thailand | Observational with AI development and validation | 500 patients (500 PRs; 14,997 teeth; 1,792 caries) | ≥13 years; females mean 25.7, males 26.2 | Dental caries (enamel, dentine, pulp involvement) | YOLOv5 (tooth detection), Attention U-Net (caries segmentation) | PRs with bitewing confirmation | Ground truth by 3 radiologists consensus | AI-assisted tooth detection and caries segmentation on PRs | IoU, DSC/F1, precision, recall, specificity, accuracy, weighted kappa, Bland–Altman | Strong agreement: weighted kappa 0.943 (overall), 0.907 (enamel), 0.948 (dentine), 0.981 (pulp); IoU 0.66–0.75; DSC/F1 0.79–0.85; accuracy 0.93–0.94 surpassing previous panoramic caries models. |
| (Saber et al., 2025) | Egypt | Observational with AI development and validation | 699 patients (699 periapical radiographs); 70 external validation | 12–78 years (internal); 16–66 years (external) | Apical periodontitis scored via PAI (1–5) | YOLOv8m, YOLOv11m, YOLOv12m | Digital periapical radiographs (VistaScan, Soredex, EzSensor HD) | Ground truth by 3 calibrated examiners consensus | Automated detection and classification of apical periodontitis | Precision, Recall, F1 score, mAP50, IoU, confusion matrix, McNemar's test | mAP50: 86.4–86.6%; Precision: 86.8–89.1%; max F1: 87.1% (YOLOv11m); superior detection of PAI 3–5; external validation showed consistent performance with post-hoc power >99%. |
| (Vinayahalingam et al., 2021) | Netherlands | Retrospective pilot study | 253 PRs; 250 carious and 250 non-carious M3s | 16–80 years; mean 31.7 ± 12.7 | Dental caries in mandibular and maxillary third molars | CNN, MobileNet V2 | PRs (cropped 256×256 px images) | Ground truth from electronic medical records | Automated classification of carious lesions in third molars | Classification accuracy, sensitivity, specificity, AUC, F1-score, confusion matrix | Accuracy 0.87, sensitivity 0.86, specificity 0.88, AUC 0.90 with Grad-CAM visualization demonstrating effective automated caries classification in third molars. |
| (Wang et al., 2025) | Netherlands, Brazil, Taiwan | Retrospective, multicenter AI evaluation | 6,669 DPRs; Netherlands: 5,245, Brazil: 1,173, Taiwan: 251 | Mean 40.2 years; range 16–95 | Missing teeth, implants, residual roots, crown/bridge, root canal fillings, fillings, caries, periapical radiolucencies | Deep CNNs combining object detection and semantic segmentation | DPRs | Expert dental practitioners; 4 dentists (2 general, 2 specialists) | Automated per-tooth identification and assessment of 8 dental findings | Sensitivity, specificity, precision, AUC-ROC, F-scores, Cohen's Kappa, reading time | Macro-averaged AUC-ROC 96.2%; AI significantly improved sensitivity for periapical radiolucencies and missing teeth versus humans; reading time 1.55s vs 122s; robust generalization across datasets. |
| (Xue et al., 2024) | China | Retrospective AI evaluation | 320 patients; 8,462 teeth | Not specified | Tooth position, outline, tissue, bone loss, periodontitis stage | Deep learning ensemble: YOLOv8, Mask R-CNN, TransUNet | DPRs | 3 periodontists (ground truth) | Automated tooth detection, segmentation, and periodontal disease assessment | Bone loss deviation, PCC, ICC, diagnostic accuracy | Bone loss deviation 5.28%; PCC 0.832; ICC 0.806; total diagnostic accuracy 89.45% demonstrating high efficiency and clinical utility for rapid diagnosis. |
| (Yoon et al., 2024) | South Korea | Retrospective AI evaluation | 24,578 intraoral images | Not specified | Tooth number recognition; dental caries (location and stage) | Cascade R-CNN (deep CNN) | Full intraoral photographs (occlusal, lateral, frontal views) | Annotated ground truth (bounding boxes) | Automated tooth number recognition and dental caries detection | mAP for tooth recognition and caries detection | Tooth recognition mAP 0.880; caries detection mAP 0.769 (range 0.695–0.893) demonstrating potential for practical clinical AI-driven detection application. |
| (Zhang et al., 2024) | China | Prospective clinical study | 191 patients (4,361 teeth) | Mean 46 (range 23–86) | Dental caries | MobileNet-v3 + 5-layer U-net | Intraoral camera images (1920×1080 px) | Clinical diagnosis by endodontic specialists | AI-assisted caries detection in intraoral images | Accuracy, sensitivity, specificity, PPV, NPV, F1 score | Overall accuracy 93.40%, sensitivity 81.31%, specificity 95.65%, PPV 77.68%, NPV 96.49%, F1 0.71; highest accuracy in anterior teeth with variable sensitivity for interproximal lesions. |
| (Zhu et al., 2023) | China | Retrospective development and evaluation study | Training: 1,996 PRs; Evaluation: 282 PRs | Training mean 37 (17–83); Evaluation mean 34 (18–85) | Impacted teeth, residual roots, full crowns, missing teeth, caries | BDU-Net (teeth segmentation) + nnU-Net (disease segmentation, 4 parallel networks) | PRs (2440×1280 px, PNG) | 9 dentists of varying experience (H: >10 yrs, M: 3–10 yrs, L: <3 yrs) | AI-assisted diagnosis of multiple dental diseases on PRs | Sensitivity, specificity, Youden's index, AUC, diagnostic time | Sensitivity 0.554–0.964, specificity 0.990–0.999, Youden 0.544–0.960, AUC 0.772–0.980; best for impacted teeth and crowns, lowest for caries; diagnostic time 1.5s per PR approximately 35× faster than dentists. |

Table 3: Search Strategy

| **Database** | **Search String** | **Outcome** |
| --- | --- | --- |
| **PubMed** | ("artificial intelligence" OR "machine learning" OR "deep learning" OR "neural network*" OR "convolutional neural network*" OR CNN OR AI) AND (dentistry OR dental OR odontolog* OR orthodont* OR endodont* OR prosthodont*) AND (diagnos* OR detect* OR classification OR "decision making" OR "decision support") AND ("treatment planning" OR "clinical decision*" OR "therapeutic planning") | 935 |
| **Scopus** | ("artificial intelligence" OR "machine learning" OR "deep learning" OR "neural network*" OR "convolutional neural network*" OR CNN OR AI) AND (dentistry OR dental OR odontolog* OR orthodont* OR endodont* OR prosthodont*) AND (diagnos* OR detect* OR classification OR "decision making" OR "decision support") AND ("treatment planning" OR "clinical decision*" OR "therapeutic planning") | 960 |
| **CENTRAL (Cochrane Library)** | ("artificial intelligence" OR "machine learning" OR "deep learning" OR neural network* OR convolutional neural network* OR AI) AND (dentistry OR dental OR odontolog*) AND (diagnos* OR detect* OR "decision making" OR "decision support") AND ("treatment planning" OR "clinical decision*") | 87 |
| **Embase** | ("artificial intelligence" OR "machine learning" OR "deep learning" OR "convolutional neural network") AND (dentistry OR "dental imaging" OR "oral diagnosis") AND (diagnostic accuracy OR "decision support" OR "clinical decision making") AND ("treatment planning" OR "therapeutic planning") | 70 |
| **Web of Science** | ("artificial intelligence" OR "machine learning" OR "deep learning" OR "convolutional neural network" OR "neural network") AND (dentistry OR "dental radiology" OR "oral diagnosis") AND (diagnostic accuracy OR "clinical decision making" OR "decision support system") AND ("treatment planning" OR "dental treatment planning") | 274 |
| **Total** |  | **2326** |

Table 4: Quality Assessment using QUADAS-2

| **Study (Author, Year)** | **Domain 1: Patient Selection** |  |  |  |  | **Domain 2: Index Test** |  |  |  | **Domain 3: Reference Standard** |  |  |  | **Domain 4: Flow and Timing** |  |  |  |  |
| --- | --- | --- | --- | --- | --- | --- | --- | --- | --- | --- | --- | --- | --- | --- | --- | --- | --- | --- |
|  | **Consecutive/Random?** | **Case-control avoided?** | **Inappropriate exclusions avoided?** | **Risk of bias? (Patient Selection)** | **Applicability concerns? (Patient Selection)** | **Interpreted blind?** | **Threshold pre-specified?** | **Risk of bias? (Index Test)** | **Applicability concerns? (Index Test)** | **Reference standard appropriate?** | **Interpreted blind? (Reference)** | **Risk of bias? (Reference)** | **Applicability concerns? (Reference)** | **Appropriate interval?** | **All received reference?** | **Same reference?** | **All in analysis?** | **Risk of bias? (Flow & Timing)** |
| (Alotaibi et al., 2022) | Unclear | Yes | Yes | Unclear | No | Yes | Yes | No | No | Yes | Yes | No | No | Yes | Yes | Yes | Yes | No |
| (Bayati et al., 2025) | Unclear | Yes | Yes | Unclear | No | Yes | Yes | No | No | Yes | Yes | No | No | Yes | Yes | Yes | Yes | No |
| (Çelik & Çelik, 2022) | Unclear | Yes | Unclear | Unclear | No | Unclear | Unclear | Unclear | No | Unclear | Unclear | Unclear | No | Yes | Yes | Yes | Yes | Unclear |
| (Chau et al., 2025) | Unclear | Yes | Yes | Unclear | No | Unclear | Unclear | Unclear | No | Yes | Unclear | Unclear | No | Yes | Yes | Yes | Yes | Unclear |
| (Esmaeilyfard et al., 2023) | Unclear | Yes | Yes | Unclear | No | Yes | Yes | No | No | Yes | Yes | No | No | Yes | Yes | Yes | Yes | No |
| (Frutos et al., 2024) | Yes | Yes | Yes | No | No | Yes | Yes | No | No | Yes | Yes | No | No | Yes | Yes | Yes | Yes | No |
| (Kazimierczak et al., 2024) | Unclear | Yes | Yes | Unclear | No | Yes | Yes | No | No | Yes | Yes | No | No | Yes | Yes | Yes | Yes | No |
| (Leemput et al., 2024) | Unclear | Yes | Yes | Unclear | No | Yes | Unclear | Unclear | No | Yes | No | No | No | Yes | Yes | Yes | Yes | No |
| (Pérez et al., 2023) | Yes | Yes | Yes | No | No | Yes | Yes | No | No | Yes | Yes | No | No | Yes | Yes | Yes | Yes | No |
| (Pornprasertsuk-Damrongsri et al., 2025) | Unclear | Yes | Yes | Unclear | No | Yes | Yes | No | No | Yes | Yes | No | No | Yes | Yes | Yes | Yes | No |
| (Saber et al., 2025) | Unclear | Yes | Yes | Unclear | No | Yes | Yes | No | No | Yes | Yes | No | No | Yes | Yes | Yes | Yes | No |
| (Wang et al., 2025) | Unclear | Yes | Yes | Unclear | No | Yes | Unclear | Unclear | No | Yes | Unclear | Unclear | No | Yes | Yes | Yes | Yes | Unclear |
| (Xue et al., 2024) | Unclear | Yes | Yes | Unclear | No | Yes | Unclear | Unclear | No | Yes | Unclear | Unclear | No | Yes | Yes | Yes | Yes | Unclear |
| (Yoon et al., 2024) | Unclear | Yes | Yes | Unclear | No | Yes | Unclear | Unclear | No | Yes | Unclear | Unclear | No | Yes | Yes | Yes | Yes | Unclear |
| (Zhang et al., 2024) | Yes | Yes | Yes | No | No | Yes | Unclear | Unclear | No | Yes | Unclear | Unclear | No | Yes | Yes | Yes | Yes | Unclear |
| (Zhu et al., 2023) | Unclear | Yes | Yes | Unclear | No | Yes | Yes | No | No | Yes | No | No | No | Yes | Yes | Yes | Yes | No |
